# Prevalence and Correlates of Asymptomatic Malaria and Anemia on First Antenatal Care Visit among Pregnant Women in Southeast, Tanzania

**DOI:** 10.3390/ijerph17093123

**Published:** 2020-04-30

**Authors:** Eulambius M. Mlugu, Omary Minzi, Appolinary A. R. Kamuhabwa, Eleni Aklillu

**Affiliations:** 1 Division of Clinical Pharmacology, Department of Laboratory Medicine, Karolinska Institutet at Karolinska, University Hospital, Huddinge, 141 86 Stockholm, Sweden; mlugusonlove@gmail.com; 2 Department of Pharmaceutics, School of Pharmacy, Muhimbili University of Health and Allied Sciences, Dar es Salaam 0702172, Tanzania; 3 Department of Clinical Pharmacy and Pharmacology, School of Pharmacy, Muhimbili University of Health and Allied Sciences, Dar es Salaam 0702172, Tanzania; minziobejayesu@gmail.com (O.M.); enali2012@gmail.com (A.A.R.K.)

**Keywords:** pregnancy, pregnant women, malaria, asymptomatic malaria, anemia, antenatal care

## Abstract

Asymptomatic malaria and anemia during pregnancy increase the risk of negative birth outcomes. This cross-sectional study investigated the prevalence and correlates of asymptomatic malaria and anemia during first antenatal care (ANC) visit among pregnant women in a rural district, Tanzania. HIV-uninfected pregnant women without symptoms of malaria (*n* = 819) attending their first ANC at Kibiti Health Centre were enrolled from February 2017 to February 2018. Asymptomatic malaria was detected by malaria rapid-diagnostic tests (mRDT) and real-time PCR. Hemoglobin concentration was determined by HemoCue Hemoglobin 201+. The study outcomes were the prevalence of asymptomatic malaria and anemia (Hemoglobin level <11 g/dL). The overall prevalence of asymptomatic malaria was 36.4% (95% CI: 33.1, 39.8). The monthly prevalence of asymptomatic malaria remained >25% throughout the year, and the highest prevalence (40%) was recorded during the rainy season. Asymptomatic malaria was significantly associated with primigravida, younger maternal age, and anemia. The prevalence of anemia was 68.5% (95% CI: 65.2, 71.6). Asymptomatic malaria, primigravida, younger maternal age and low Body Mass Index were significant predictors of low hemoglobin concentration. We report high prevalence of asymptomatic malaria and anemia among pregnant women on the first ANC visit. Screening of malaria and anemia during the first ANC visit is recommended for targeted interventions.

## 1. Introduction

Malaria and anaemia in pregnancy are significant maternal health problems causing an increased risk for maternal death, stillbirth, spontaneous abortion, and low birth weight [1,2]. The World Health Organization (WHO) recommends antenatal care (ANC) involving intermittent preventive treatment in pregnancy with sulfadoxine-pyrimethamine (IPTp-SP) and folic acid supplementation for prevention of malaria and anemia respectively in all pregnant women living in endemic countries. However, in most cases, pregnant women begin attending ANC after the first trimester has passed, and therefore, do not fully benefit from the service [3,4]. Thus, evaluating the efficiency of existing interventions for prevention of malaria and anemia provides valuable information for better planning and implementation of public health programs. 

In low-malaria-transmission areas, there is poor acquired immunity; hence, severe symptomatic episodes of malaria in pregnancy are common, requiring prompt diagnosis and treatment [5]. On the other hand, due to acquired immunity in moderate and high malaria transmission areas, malaria is associated with asymptomatic cases. Asymptomatic infections present no clinical manifestation, thus remain undetected and untreated. Malaria-infected erythrocytes may accumulate in the placenta causing adverse birth outcomes even in undetected peripheral parasitemia [6,7]. Asymptomatic malaria leads to infected erythrocytes sequestering within the placenta. The sequestration and adherence of *P. falciparum-*infected erythrocytes to the placenta is mediated by variant surface antigen 2-CSA (var2CSA), a member of *P. falciparum* erythrocyte membrane protein 1 (*Pf*EMP1) which binds specifically to chondroitin sulfate A in placental cells [8]. Placental malaria infection is associated with preterm delivery, intrauterine growth restriction, stillbirths, low birth weight (LBW), and neonatal deaths [6,9]. 

Asymptomatic malaria in pregnancy remains an important contributor of maternal anemia, the leading cause of malaria related morbidity and mortality. Additionally, asymptomatic malaria may serve as parasite reservoirs contributing to the cycle of malaria transmission [10,11]. The World Health Organization (WHO) recognizes asymptomatic malaria as one of the challenges for malaria elimination, and suggest that control strategies should take into account this infectious parasite reservoir [12]. The WHO recommends a protection package against malaria for all pregnant women living in endemic areas. The package includes sleeping under insecticide-treated nets, indoor spraying, effective case malaria management and intermittent preventive treatment in pregnancy with sulphadoxine-pyrimethamine (IPTp-SP) [13]. Tanzania, like many other sub-Sahara African countries, has adopted and integrated the malaria control strategies recommended by WHO. IPTp involves provision of monthly single dose of sulfadoxine-pyrimethamine (SP) during ANC visit starting early in the second trimester. At least three doses (≥3 SP) of IPTp-SP given monthly is considered optimum during pregnancy [14]. In Tanzania, 26% of eligible pregnant women received optimal (≥3) IPTp-SP and 56% receive at least 2 doses of IPTp-SP in 2017 [15]. Late initiation of ANC among pregnant women is one of the reasons for low coverage of optimal IPTp-SP in Tanzania [16]. On the other hand, the reported high levels of falciparum dihydrofolate reductase (*Pfdhfr*) and dihydropteroate synthetase (*Pfdhps*) mutations and associated haplotype conferring SP resistance in Tanzania may undermine the use of IPTp-SP in improving pregnancy outcomes [17]. 

The Tanzanian malaria control program strives to increase coverage of insecticide-treated bed nets (ITNs), and promote effective case management as strategies to control malaria among pregnant women before they begin ANC where they receive IPTp-SP [18]. Since malaria in pregnancy is usually asymptomatic, ITNs are the main strategy for malaria control in pregnant women before and after initiation of IPTp-SP. ITNs protect against malaria both by acting as a physical barrier between mosquitoes and humans, and by repelling or killing susceptible mosquitoes. In Tanzania, mass distribution campaign and the national voucher scheme [19] have largely contributed to improved ITNs coverage to 78% in 2018 [20]. According to the recent Tanzania malaria indicator survey, the overall ITN coverage has increased to 78% [15]. However, the question remains to what extent these strategies protect pregnant women who are vulnerable to malaria before they begin ANC. Assessment of asymptomatic malaria at first antenatal clinic visit is important to evaluate the efficiency of these interventions in protecting women against malaria before the initiate of IPTp-SP. 

The prevalence, severity, risk factors and treatment outcome of symptomatic malaria among pregnant women in Tanzania is investigated previously [9,21,22,23]. However, data on the prevalence of asymptomatic malaria and the burden of anemia among pregnant women who are not yet enrolled in the ANC are scarce in most sub-Sahara African countries including Tanzania. Evaluating the burden of asymptomatic malaria and anemia in pregnant women, particularly in high malaria transmission areas, is important to inform policymakers for targeted interventions, effective planning and implementation of malaria control programs. Therefore, this study investigated the timing of first attendance to the antenatal clinic, the burden of asymptomatic malaria and anemia, and associated risk factors at first antenatal care visits among pregnant women living in a moderate malaria transmission area in Tanzania. 

## 2. Materials and Methods 

### 2.1. Study Design and Population

This was a healthcare facility-based cross-sectional study that targeted HIV-negative pregnant women attending their first ANC. Data collection took place from February 2017 to February 2018 and focused mainly on investigating the prevalence of asymptomatic malaria, anemia, and timing for the first ANC. Inclusion criteria were pregnant women who visited ANC for the first time, HIV negative, and no symptoms of malaria. The exclusion criteria were; pregnant women attending ANC for the second time or more, those started on IPTp-SP, those with a history of malaria and presumptively treated for the past one month and those who presented with symptoms related to malaria (fever, headache, chills, body ache, and joints ache). One month’s history of malaria was chosen as an exclusion criterion because the target antigen (HRP2) for mRDT would still react positively for that duration after an acute malaria episode. Participants who met inclusion criteria and consented to participate were enrolled.

### 2.2. Study Site

**Figure 1:**
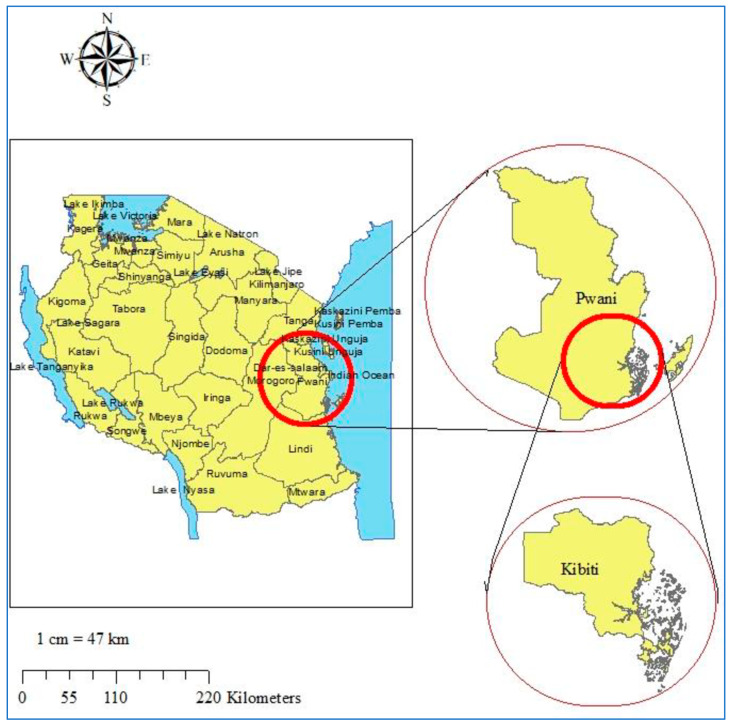
Study site map. Top right is the map of Coast region. Bottom right is the map of Kibiti district. On the left is the map of Tanzania. The study site map was originally generated using ArcGIS software version 10.7.1 (Esri, California, USA; https://www.esri.uconn.edu/software/arcgis-student/).

The study was conducted at Kibiti Health Centre in Kibiti District. The district is located in the coast region of southeast Tanzania (Figure 1). The area lies south of the country’s business city (Dar es Salaam) extending between 7.7218° S and 38.9375° E along the Dar es Salaam-Lindi and Mtwara highway. The district is part of hot, humid, coastal plain with varying tropical climatic conditions. The study area is characterized by seasonal rainy and vast water bodies which facilitate transmission of malaria. The region normally gets rain twice a year; the short rainy season occurs during September and October, and the longest rainfall happens from February to May. Malaria is a major public health problem in the area, accounting for the majority of out-patient health facility attendance. Malaria transmission is still endemic and common during and after the period of long rain. About 96% of all malaria cases are caused by *Plasmodium falciparum,* while 4% are due to *P. malariae* and *P. ovale* [24]. The prevalence of malaria in the general population is 13%, according to the epidemiological characterization of malaria in the area [25].

### 2.3. Data Collection Procedures

Socio-demographics data, medical and obstetric history, the use of medication and insecticide-treated bed nets were collected using an individual case record format. Gestational age was determined by the last menstrual period (LMP) and the fundal height method, which is the standard of care in Tanzania. Weight was measured by digital weighing scale in kilograms (nearest 0.1 kg). Height was measured by a potable wooden scale in centimeter (nearest 0.1 cm). Temperature was recorded from the maternal armpit using a digital thermometer and fever was defined as a temperature of ≥37.5 °C. All recruited pregnant women were tested for HIV sero-status as a part of a routine ANC procedure. Peripheral finger-prick blood was collected for malaria detection and hemoglobin concentration determination. Asymptomatic malaria was detected by RDT (Care start, ACCESS BIO Somerset, NJ, USA) *and* real-time PCR (Applied Biosystems). Hemoglobin concentration was determined by HemoCue Hemoglobin 201+ analyzer (HemoCue AB Angelholm, Sweden).

### 2.4. Detection of Malaria Parasites by Rapid Diagnostic Test

Peripheral finger-prick blood was used to screen for malaria using Malaria rapid diagnostic tests (mRDTs). Malaria Pf/PAN (HRP2/PLDH) Ag combo RDTs (Care start, ACCESS BIO Somerset, NJ U.S.A) were used according to the manufacturer’s instructions, and results were examined after 20 min following the addition of four drops of the wash buffer. The test detects four *Plasmodium* species responsible for malaria in human (*P. falciparum, P. ovale, P. vivax and P. malariae)*. RDTs target *P. falciparum* antigens histidine-rich protein 2 (HRP2) and *Plasmodium* lactate dehydrogenase (pLDH) for *P. falciparum, P. ovale, P. vivax* and *P. malariae.*

### 2.5. Detection of Malaria Parasites by Real-Time PCR

Finger-prick blood samples were collected on filter papers and stored dried. Three circles of 3 mm each in diameter were punched from dried blood spots on filter papers to extract DNA using QIAamp DNA blood micro kit (Qiagen GmbH, Hilden, Germany) according to the manufacturer’s instructions. Screening for Plasmodium parasite species (*P. falciparum, P. vivax, P. ovale, and P. malariae*) was performed using a species-specific multiplex real-time PCR assay targeting the ssRNA gene as described previously [21,26]. The master mix for a single reaction included species-specific probes and forward primers for all four *Plasmodium* species used in combination with a conserved reverse primer. The *P. ovale-, P. malariae-, P. vivax-* and *P. falciparum*-probes (Applied Biosystems) were each labelled with a distinct fluorophore and Mustang Purple was used as the reference dye. The reaction was performed in duplicate in a final volume of 15 μL per well containing 3 μL DNA, 7.5 μL of TaqMan multiplex master mix (Applied Biosystems), 0.3 μL (10 μmol/L) of each species-specific forward primers, 0.75 μL (10 μmol/L) of the reverse primer, 0.15 μL (10 μmol/L) of each species-specific probe, passive reference dye Mustang Purple and DNA/RNA-free water. All reactions were run on 7500 Fast Real-Time PCR system (Applied Biosystems) with the following settings; each sample was initially denatured at 95 °C for 20 s and cycled 45 times, with each cycle consisting of 95 °C for 3 s and 60 °C for 30 s. Each reaction plate included four positive controls for *P. falciparum*, *P. ovale*, *P. vivax*, and *P. malariae* and a negative control with molecular-grade water instead of DNA.

### 2.6. Determination of Hemoglobin Concentration

Hemoglobin level was determined from peripheral finger prick blood by using digital HemoCue Hemoglobin 201+ analyzer (HemoCue AB Angelholm, Sweden) and expressed as g/dL. Anemia was defined when maternal hemoglobin level was <11 g/dL. Mild, moderate and severe anemia were defined when maternal hemoglobin levels were 10–10.9 g/dL, 7–9.9 g/dL and <7 g/dL, respectively according to the WHO guideline on the use and interpretation of haemoglobin concentrations for diagnosis of anaemia and assessment of severity in pregnant women [27]. 

### 2.7. Study Outcomes

The primary study outcomes were the prevalence of asymptomatic malaria and anemia (Hemoglobin level <11 g/dL) among pregnant women at first ANC visit. Secondary outcomes were the severity of anemia according to WHO classification (mild anemia hemoglobin level, 10–10.9 g/dL; moderate anemia, hemoglobin level 7–9.9 g/dL and severe anemia hemoglobin level <7 g/dL) [27]. Factors associated with malaria and anemia were determined. 

### 2.8. Data Management and Analysis

Data were entered into Microsoft Excel and transferred to Statistical Package for Social Sciences (SPSS) version 20 software (IBM Corp, New York, USA) for analysis. Descriptive analysis used *n* (%) for binary and categorical variables and mean (SD) with 95% CI or median (minimum and maximum) for continuous variables. Normality was assumed and checked for all continuous variables. The Shapiro–Wilk test *p* values for all independent variables were >0.05. Visual inspection of histograms, normal Q-Q plots, and box plots showed that hemoglobin was approximately normally distributed for all independent variables with skewness and kurtosis values with their respective standard errors were all close to zero (between −1 and +1). Linear regressions were used to analyze independent predictors of hemoglobin concentration. Asymptomatic malaria was defined as having a positive mRDT and/or detection of malaria parasites using real-time PCR in the absence of malaria symptoms. Anemia was defined as having hemoglobin concentration below 11 g/dL. Binary logistic regressions were used to determine the predictors of asymptomatic malaria and anemia among pregnant women. Variables with a *p*-value ≤ 0.2 in the univariate analysis were included in the multivariate model. The goodness of fit of the model was checked by Hosmer and Lemeshow test (*p* > 0.05). Crude and adjusted odds ratios with their respective *p* values were presented. Significance level was set at 0.05 and the confidence level at 95%. All *p* values were two-sided, and a *p* value of less than or equal to 0.05 was considered to indicate statistical significance. 

### 2.9. Ethics Approval 

The study was ethically approved by the Institutional Review Board of the National Institute for Medical Research (NIMR), Tanzania (NIMR/HQ/R.8a/Vol.IX/2342), and Muhimbili University of Health and Allied Sciences (MUHAS) Tanzania (2016-06-07/AEC/Vol.XI/2). Written informed consent was obtained from all study participants before commencement of the study.

## 3. Results

**Figure 2:**
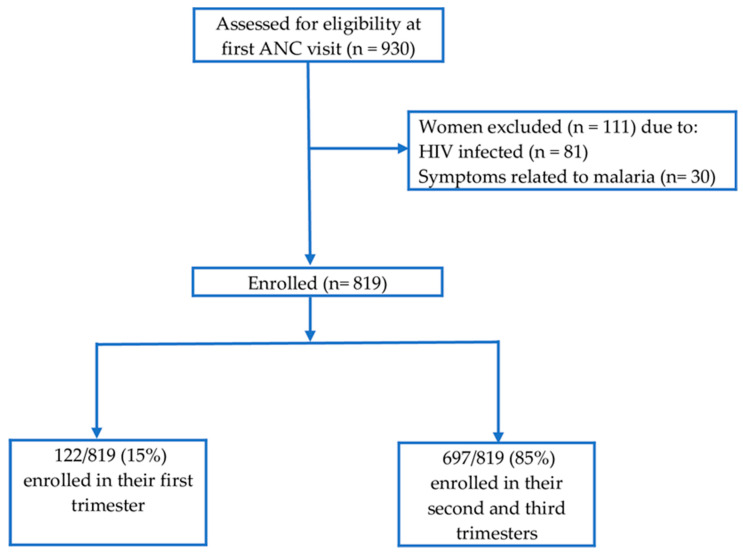
Participants’ recruitment flow chart.

From February 2017 to February 2018, a total of 930 pregnant women attending their first ANC were screened for eligibility and 819 pregnant women were enrolled (Figure 2). The average gestational age at first ANC visit was 5.4 months. Eighty-five percent of enrolled participants started their first ANC visit in their second and third trimesters. Enrolled participants were mainly young adult women aged 20–34 years. About half (51.2%) of enrolled women were pregnant for the third time and above (multigravida). The majority of enrolled women (80.1%) reported to be using insecticide treated bed nets. Socio-demographic and obstetric characteristics of the study participants are summarized in Table 1. 

### 3.1. Prevalence of Asymptomatic Malaria at First Antenatal Care Visit

**Figure 3:**
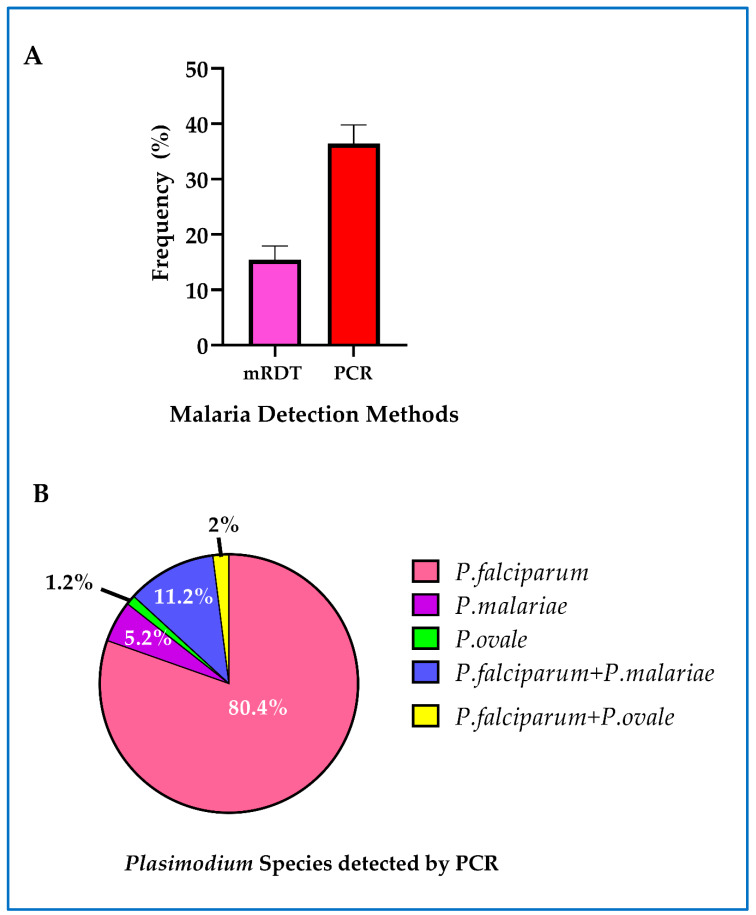
Asymptomatic malaria among study participants: (**A**) overall prevalence of asymptomatic malaria detected by mRDT, real-time PCR and combined mRDT and PCR. The error bars represent 95% confidence intervals; (**B**) the distribution of Plasmodium malaria parasite species detected by real-time PCR.

Asymptomatic malaria was detected using mRDT and real-time PCR. The overall prevalence of asymptomatic malaria at first ANC visit detected by real-time PCR was 36.4% [298/819] (95% CI = 33.1 to 39.8). On the other hand, the prevalence of asymptomatic malaria detected by mRDT alone was 15.4% [126/819] (95% CI: 12.9 to 17.9) (Figure 3A). The sensitivity and specificity of mRDT compared to real-time PCR were 43.8% and 98.7%, respectively. *P. falciparum* mono-infection was the most predominant detected in 80.4% (202/251) of women who were positive by real-time PCR. Mixed infection of *P. falciparum* with *P. malariae* and *P. ovale* were detected in 11.2% (28/251) and 2% (5/251), respectively. Mono-infection with *P. malariae* and *P. ovale* were detected in 5.2% (13/251) and 1.2% (3/251) women respectively (Figure 3B). *P. vivax* infection was not detected.

**Figure 4:**
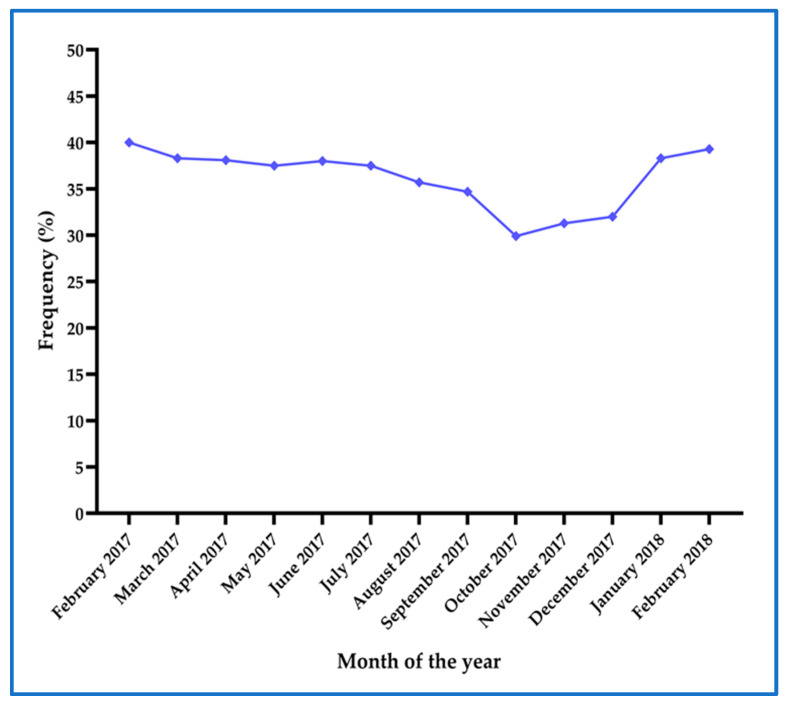
Prevalence of asymptomatic malaria as determined by combined mRDT and real-time PCR at each month throughout the study period.

The monthly prevalence of asymptomatic malaria determined by mRDT and real-time PCR at first ANC visit remained above 25% throughout the year (Figure 4) from February 2017 to February 2018, with higher prevalence recorded during the rainy season (February to May)

### 3.2. Factors Associated with Asymptomatic Malaria 

The association of asymptomatic malaria with various potential correlates was evaluated. Binary logistic regression analysis indicated that asymptomatic malaria was significantly associated with gravidity, maternal age, and anemia. Primigravida was associated with significantly higher odds of asymptomatic malaria compared to multigravida (Table 2). In addition, adolescence (age <20 years) was significantly associated with asymptomatic malaria compared to adults (age ≥20 years). Moreover, asymptomatic malaria was significantly associated with moderate to severe anemia (Table 2). 

### 3.3. Prevalence of Anemia at First Antenatal Care Visit

**Figure 5:**
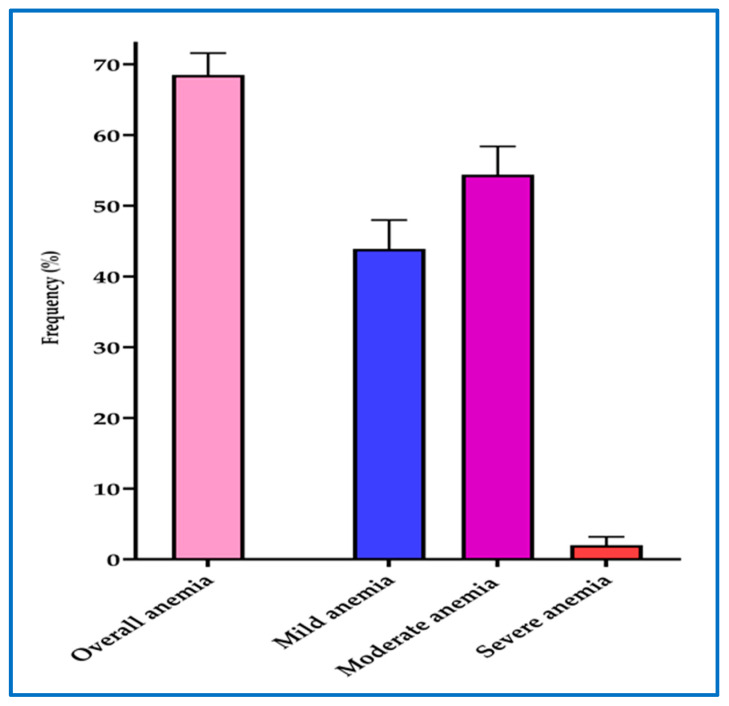
Overall prevalence and severity of anemia among pregnant women. The error bars represent 95% confidence intervals. Mild anemia (Hb = 10–10.9 g/dL), Moderate anemia (Hb = 7–9.9 g/dL), Severe anemia (Hb < 7 g/dL).

The overall prevalence of anemia among pregnant women at first ANC visit was 68.5% [561/819] (95% CI = 65.2 to 71.6). Majority of participants (54.4% [305/561]) had moderate anemia (hemoglobin = 7–9.9 g/dL) followed by mild anemia 43.9% [246/561] (hemoglobin = 10–10.9 g/dL) and only a few pregnant women (1.7% [10/561]) had severe anemia (hemoglobin <7 g/dL) (Figure 5).

### 3.4. Factors Associated with Anemia 

Potential predictors of anemia at first ANC visit were investigated using binary logistic regression. Maternal age, gravidity, and late initiation of ANC (gestational age ≥21 weeks) were significantly associated with anemia at first ANC visit. Adolescence (age <20 years) was significantly associated with higher odds of anemia compared to adults (age ≥20 years) (Table 3). On the other hand, primigravida was significantly associated with higher odds of anemia compared to multigravida. In the multivariate binary logistic model, primigravida and late attendance to ANC (gestational age ≥21 weeks) were significantly associated with higher odds of anemia (Table 3).

### 3.5. Factors Associated with Hemoglobin Concentration

The overall mean hemoglobin concentration with one standard deviation was 10.3 (1.4) g/dL. Various factors associated with hemoglobin concentration were evaluated using linear regression model. Hemoglobin concentration was significantly associated with asymptomatic malaria, gravidity, body mass index (BIM) and maternal age (Table 4). Asymptomatic malaria was significantly associated with reduced hemoglobin concentration. In addition, multigravida was associated with significantly higher hemoglobin concentration compared to primigravida. Moreover, an increase in one unit (kg/m^2^) BMI and an increase in maternal age by one year were significantly associated with increased hemoglobin concentrations (Table 4). In the multivariate regression model, primigravida and low BMI were significantly associated with low hemoglobin concentration (Table 4).

## 4. Discussion

This study assessed the prevalence and risk factors of asymptomatic malaria and anemia at first ANC visit among HIV negative pregnant women living in a moderate malaria transmission area of Tanzania. The major findings include: (i) a high prevalence of asymptomatic malaria (36.4%) at the first antenatal care visit, which sustained to be >25% throughout the year during the study period, (ii) an overall high prevalence of anemia (>60%), (iii) delayed presentation for the first antenatal care (5.4 months) and (iv) primigravida and younger maternal age as risk factors for asymptomatic malaria, and anemia at first ANC visit. The prevalence of asymptomatic malaria (36.4%) at first ANC before initiating IPTp-SP found among pregnant women in the study area is higher than the prevalence of asymptomatic malaria at first ANC reported from Kenya (35%) [28], but lower than that reported in Uganda (50%) [29] and Malawi (54.2%) [30]. The difference in the burden of asymptomatic malaria at first ANC visit could be due to different malaria transmission intensities between different countries. Nevertheless, this finding provides further evidence that asymptomatic malaria is still a public health problem among pregnant women in endemic countries. 

The high prevalence of asymptomatic malaria among pregnant women found in this study could indicate the challenge with the current malaria prevention strategies during early pregnancy before initiating the IPTp-SP. In Tanzania, the national malaria control program strives to expand ITNs coverage which is the key strategy to prevent malaria. However, utilization of ITNs among pregnant women in Tanzania has declined from 75% in 2011 to 51% in 2017 [15,31]. While the impact of INTs in preventing malaria in sub-Saharan Africa is well known [32], the declining trend in its usage among pregnant women and outdoor mosquito biting could be the reasons for the detected high prevalence of asymptomatic malaria among pregnant women in the study area. On the other hand, factors such as poor sanitation and agricultural activities commonly practiced along water sources may favor breeding of vectors, thus facilitating malaria transmission in the setting. The use of mass media to improve awareness, especially on behavior change related to sanitation and ITNs use may help to reduce the burden. Other strategies such as indoor residual spray have been reported to be effective for the prevention of malaria in sub-Saharan Africa [33].

The prevalence of asymptomatic malaria among pregnant women during their first ANC visit was consistently above 25% throughout the study year. The highest prevalence (40 %) was recorded in February, indicating high transmission during the rainy season. The persistent prevalence of asymptomatic malaria suggests the stable transmission in the study area. The overall prevalence of asymptomatic malaria in pregnant women found in this study is five-fold higher than the current overall prevalence of symptomatic malaria in Tanzania 7.3% [20]. Currently, the national ANC protocol recommends the use of IPTp-SP for all asymptomatic pregnant women from the early second trimester and effective symptomatic case treatment. However, the high burden of asymptomatic malaria found in this study indicate the need to integrate screening and treatment of asymptomatic malaria at first ANC which is currently not commonly practiced in the study area. The prevalence of asymptomatic malaria determined by the current diagnostic standard of care in Tanzania (RDTs) (15.4%) was also two-fold higher than the national prevalence of symptomatic malaria [20]. This finding suggests that despite the limited sensitivity of mRDT compared to PCR, the use of RDTs for screening and treatment at first ANC could help to reduce the burden of asymptomatic malaria among pregnant women. 

In sub-Saharan Africa, malaria continues to be the main reason for low birth weight which is the risk factor for poor neurodevelopment and infant mortality [34]. For instance, in 2018, 16% of all children born with LBW were associated to malaria exposure during pregnancy [35]. Exposure to asymptomatic malaria during the second and third trimesters may result in placental infection and leading to LBW [36]. Thus, the high burden of asymptomatic malaria found in this study suggests the need to strengthen control of malaria in pregnancy in order to improve maternal and newborn health. The study results indicate that primigravida and adolescent pregnant women were more vulnerable to asymptomatic malaria. The reason for primigravida having a higher risk and burden of asymptomatic malaria than multigravida could be related to parity level immunity to variant surface antigen of *P. falciparum* erythrocyte membrane protein 1 (PfEMP1), which is acquired through consecutive pregnancies [37]. On the other hand, the significantly higher odds of asymptomatic malaria among adolescent pregnant women compared to adult women may be related to immunity associated with the increasing maternal age. 

High overall prevalence of anemia (68.5%) among pregnant women was found in this study. Majority of participants had moderate to mild anemia. Only few pregnant women were found to have severe anemia at first ANC visit. Pregnant women with asymptomatic malaria had significantly higher odds of moderate to severe anemia compared to those without detected malaria parasites. In addition, asymptomatic malaria was significantly associated with low hemoglobin concentration. A study in Cameroon, reported that both asymptomatic and symptomatic malaria at first ANC visit were associated with low hemoglobin levels and anemia [38]. Therefore, our finding adds evidence to further support the association of asymptomatic malaria with anemia during pregnancy. Malaria in pregnancy may causes anemia through hemolysis, increased splenic clearance of erythrocytes, and reduced red blood cell production [39]. Malaria associated maternal anemia is the leading cause of morbidity and mortality associated with malaria in pregnancy [35].

Factors such as poor nutrition could be among the reasons for high anemia in the study area. In this study, BMI was associated with significantly low hemoglobin concentration. Although caution is needed when relating BMI with nutritional status especially during pregnancy, this finding may suggest that poor nutrition could be one of the reasons for the high anemia burden in the study area. Additionally, primigravida and adolescent pregnant women were found to have significantly higher odds of anemia compared to multigravida and adult pregnant women, respectively. The high risk of anemia among primigravida and adolescent women may be related to their higher susceptibility to asymptomatic malaria. Anemia prevalence found in this study (68.5 %) is higher than that reported in Nigeria (59.6%), Southeast Ethiopia (27.9%) and in low malaria area of northern Tanzania (23%) [40,41,42], indicating that anemia is a major public health burden among pregnant women in the study area. 

Maternal anemia during pregnancy could lead to adverse birth outcomes such as preterm birth and LBW [43,44,45]. In addition, anemia during pregnancy may cause fetal iron deficiency which is an established risk factor for poor neurodevelopment [46]. Iron is an essential requirement in fetal synaptogenesis and myelination, thus its deficiency could be the reason for poor neurodevelopment [47,48]. Poor neurodevelopment might produce long-lasting defects in mental development and performance that may further impair child learning capacity. Strategies such as early initiation of ANC and effective control of malaria during pregnancy should be strengthened. 

The median gestational age at first ANC visit was 5.4 months. The majority of pregnant women in the study area (85%) appeared to their first ANC visit in the late second and early third trimesters, which is similar to findings from other developing countries [3,4]. Usually, visits to the ANC serve as a routine standard of care platform for delivery of IPTp-SP and iron/folic acid supplementation for prevention of malaria and anemia, respectively. Late presentation to the ANC indicates that pregnant women may not fully benefit from preventive and curative services that are provided in the ANC. Our results indicate that women who initiated ANC late (gestational age ≥21 weeks) had significantly higher burden of anemia compared to those who started ANC early (gestational age <20 weeks). This finding suggest that early initiation of ANC could help to reduce the burden of anemia. Furthermore, anemia diagnosed at the late second trimester might not be corrected by iron/folic acid supplementation to term [45]. Hence sensitization of pregnant women to begin ANC visits early in the first trimester could help to improve public health among pregnant women.

Our findings may also serve as an evaluation for the efficiency of existing interventions for the prevention of malaria and anemia before ANC initiation. The results from this study indicate the need to strengthen and expand the ITNs coverage and usage and screening for malaria and anemia at first ANC visit, particularly for those pregnant women living in a high malaria transmission area.

The study had some limitations. As it was conducted in an area of moderate malaria transmission intensity (<50%), the results of this study may not be generalized to areas of lower (<5%) and higher (>50%) malaria transmission intensities. Additionally, the study assessed only asymptomatic malaria in pregnancy. Excluding pregnant women with symptomatic malaria might be underestimating the overall burden of malaria at first ANC visit in the study area. However, the strength of this study is that the data collection spans over one year, covering both high and low malaria seasons. Moreover, the use of sensitive techniques (real-time PCR) helped to detect malaria parasitemia missed by mRDT which has limited sensitivity; thus, the findings of this study may reflect the true burden of asymptomatic malaria in the study area.

## 5. Conclusions

We report delayed presentation in seeking ANC, and a high prevalence of asymptomatic malaria and anemia among pregnant women at first ANC visit. Primigravida and younger maternal age significantly correlate with both asymptomatic malaria and anemia. Asymptomatic malaria and low BMI are significant predictors of low hemoglobin concentration. Screening for malaria and anemia, particularly in primigravida and younger mothers, during the first ANC visit is recommended for targeted interventions. Strengthening ITNs coverage and screening for malaria and anemia during first antenatal care, particularly in areas with high malaria transmission is recommended for targeted intervention and care. Sensitization for earlier ANC initiation and educating women on nutrition and diets during ANC visit is recommended to reduce the burden of anemia. In addition, new strategies to outreach pregnant women who have not yet started ANC are recommended to maximize the benefit from the service and alleviate the burden of malaria and anemia in pregnancy.

## Figures and Tables

**Table 1:** Sociodemographic and obstetric characteristics of the study participants.

Characteristics (*n* = 819)		*n*	Frequency (%)
Maternal age	Mean age (SD), years	26.5 (7.2)	
	Adolescent (<20) years	158	19.3
	Young adult (20–34) years	519	63.4
	Adult (≥35) years	142	17.3
Gravidity	Primigravida	219	26.7
	Secundigravida	181	22.1
	Multigravida	419	51.2
Parity (Number of live children)	Median (range)	2 (0–9)	
Gestational age	Early ANC (≤20) weeks	356	43.5
	Late ANC (≥21) weeks	463	56.5
Insecticide treated bed net use	YES	656	80.1
	NO	163	19.9
Height	Median (range), cm	151 (140–168)	
Weight	Median (range), kg	54 (38–99)	
Body temperature	Median (range), °C	37 (34–37.4)	
Body Mass Index (BMI)	Median (range), kg/cm^2^	23.8 (16.4–40.7)	

**Table 2:** Association between asymptomatic malaria and maternal characteristics.

Characteristic	Asymptomatic Malaria *n*/*N* (%)	Univariate Analysis		Multivariate Analysis	
		OR (95% CI)	*p* Value	aOR (95% CI)	*p* Value
Age			0.005		0.09
Adolescent (16–19 years)	73/158 (46.2)	1.66 (1.17, 2.37)		1.48 (0.94, 2.35)	
Adult (≥20 years)	225/661 (34)	1		1	
Gravidity					0.06
Primigravida	94/219 (42.9)	1.46 (1.06, 2.00)	0.019	0.92 (0.85, 1.00)	
Multigravida	204/600 (34)	1		1	
Gestational age (Weeks)					
Early attendance to ANC (≤20 weeks)	135/356 (37.9)	1.12 (0.84, 1.50)	0.42		
Late attendance to ANC (≥21 weeks)	163/463 (35.2)	1			
ITN use					
YES	234/656 (35.7)	0.85 (0.60, 1.21)	0.36		
NO	64/163 (39.2)	1			
* BMI median (range) kg/m^2^	23.8 (16.4–40.7)	0.98 (0.94, 1.02)	0.31		
Anemia					
Severe & moderate anemia	129/315 (41)	1.49 (1.05, 2.10)	0.024	1.39 (0.98, 1.97)	0.07
Mild anemia	87/246 (35.4)	1.17 (0.81, 1.70)	0.40	1.13 (0.78, 1.64)	0. 51
No anemia	82/258 (31.8)	1		1	

BMI = Body mass index; ITN = insecticide treated bed net; CI = Confidence Interval; * Continuous variable; OR = crude odds ratio; aOR = adjusted odds ratio.

**Table 3:** Factors associated with anemia among pregnant women.

Characteristic	Anemia *n*/*N* (%)	Univariate Analysis		Multivariate Analysis	
		OR (95% CI)	*p* Value	aOR (95% CI)	*p* Value
Age					
Adolescent (16–19 years)	127/158 (80.4)	2.14 (1.40, 3.28)	<0.001	1.35 (0.78, 2.32)	
Adult (≥20 years)	434/661 (65.7)	1		1	0.28
Gravidity					
Primigravida	176/219 (80.4)	2.29 (1.57, 3.32)	<0.001	1.96 (1.22, 3.13)	0.005
Multigravida	385/600 (64.4)	1		1	
Gestational age					
Early attendance to ANC (≤20 weeks)	35/356 (66)	0.82 (0.61, 1.10)	0.18	0.72 (0.53, 0.98)	0.035
Late attendance to ANC (≥21 weeks)	326/463 (70.4)	1		1	
ITN use					
YES	448/656 (68.3)	0.96 (0.67, 1.39)	0.84		
NO	113/163 (69.3)	1			
* BMI median (range) kg/m^2^	23.8 (16.4–40.7)	0.97 (0.93, 1.01)	0.11	0.97 (0.93, 1.01)	0.19

BMI = Body mass index; ITN = insecticide treated bed net; CI = confidence Interval; * Continuous variable; OR = crude odds ratio; aOR = adjusted odds ratio.

**Table 4:** Linear regression analysis for determinants of hemoglobin concentration (g/dL) among pregnant women.

Characteristic	Univariate Analysis			Multivariate Analysis		
	Beta Coefficient [95% CI]	*t*	*p* Value	Beta Coefficient [95% CI]	*t*	*p* Value
Age (Years)	0.03 (0.02, 0.04)	4.36	<0.001	0.01 (−0.01, 0.02)	0.88	0.38
* Gravidity			<0.001			
Multigravida	0.64 (0.42,0.86)	5.77		0.53 (0.27, 0.80)	3.95	<0.001
Primigravida	1			1		
Gestational age (Weeks)	−0.01 (−0.03, 0.02)	−0.27	0.79			
* ITN use			0.96			
YES	1					
NO	−0.01 (−0.25,0.24)	−0.05				
BMI	0.04 (0.01, 0.07)	3.05	0.002	0.03 (0.01, 0.06)	2.49	0.013
* Asymptomatic Malaria			0.025			
YES	−0.23 (−0.44, −0.03)			−0.17 (−0.37, 0.03)		
NO	1	−2.25		1	1.65	0.09

BMI = Body mass index; ITN = insecticide treated bed net; CI = Confidence Interval; * Categorical variable; *t* = student *t*-test.
